# Transperineal vs transrectal magnetic resonance and ultrasound image fusion prostate biopsy: a pair-matched comparison

**DOI:** 10.1038/s41598-023-40371-7

**Published:** 2023-08-18

**Authors:** Masatomo Kaneko, Luis G. Medina, Maria Sarah L. Lenon, Sij Hemal, Aref S. Sayegh, Donya S. Jadvar, Lorenzo Storino Ramacciotti, Divyangi Paralkar, Giovanni E. Cacciamani, Amir H. Lebastchi, Bodour Salhia, Manju Aron, Michelle Hopstone, Vinay Duddalwar, Suzanne L. Palmer, Inderbir S. Gill, Andre Luis Abreu

**Affiliations:** 1 Center for Image-Guided Surgery, Focal Therapy and Artificial Intelligence for Prostate Cancer, USC Institute of Urology, 1441 Eastlake Ave, Suite 7416, Los Angeles, CA 90089 USA; 2 Department of Urology, Graduate School of Medical Science, Kyoto Prefectural University of Medicine, Kyoto, Japan; 3 Department of Pathology, Keck School of Medicine, University of Southern California, Los Angeles, CA USA; 4 Dornsife School of Letters and Science, University of Southern California, Los Angeles, CA USA; 5 Department of Radiology, Keck School of Medicine, University of Southern California, Los Angeles, CA USA; 6 Department of Translational Genomics, Keck School of Medicine, University of Southern California, Los Angeles, CA USA; 7 Norris Comprehensive Cancer Center, Keck School of Medicine, University of Southern California, Los Angeles, CA USA

**Keywords:** Cancer, Diseases, Medical research, Oncology, Urology

## Abstract

The objective of this study was to compare transperineal (TP) versus transrectal (TR) magnetic resonance imaging (MRI) and transrectal ultrasound (TRUS) fusion prostate biopsy (PBx). Consecutive men who underwent prostate MRI followed by a systematic biopsy. Additional target biopsies were performed from Prostate Imaging Reporting & Data System (PIRADS) 3–5 lesions. Men who underwent TP PBx were matched 1:2 with a synchronous cohort undergoing TR PBx by PSA, Prostate volume (PV) and PIRADS score. Endpoint of the study was the detection of clinically significant prostate cancer (CSPCa; Grade Group ≥ 2). Univariate and multivariable analyses were performed. Results were considered statistically significant if p < 0.05. Overall, 504 patients met the inclusion criteria. A total of 168 TP PBx were pair-matched to 336 TR PBx patients. Baseline demographics and imaging characteristics were similar between the groups. Per patient, the CSPCa detection was 2.1% vs 6.3% (p = 0.4) for PIRADS 1–2, and 59% vs 60% (p = 0.9) for PIRADS 3–5, on TP vs TR PBx, respectively. Per lesion, the CSPCa detection for PIRADS 3 (21% vs 16%; p = 0.4), PIRADS 4 (51% vs 44%; p = 0.8) and PIRADS 5 (76% vs 84%; p = 0.3) was similar for TP vs TR PBx, respectively. However, the TP PBx showed a longer maximum cancer core length (11 vs 9 mm; p = 0.02) and higher cancer core involvement (83% vs 65%; p < 0.001) than TR PBx. Independent predictors for CSPCa detection were age, PSA, PV, abnormal digital rectal examination findings, and PIRADS 3–5. Our study demonstrated transperineal MRI/TRUS fusion PBx provides similar CSPCa detection, with larger prostate cancer core length and percent of core involvement, than transrectal PBx.

## Introduction

Diagnosis of prostate cancer (PCa) diagnosis relies on transperineal (TP) or transrectal (TR) needle biopsy of the prostate followed by prostatic tissue histological evaluation. More recently, magnetic resonance imaging (MRI) and transrectal ultrasound (TRUS) fusion prostate biopsy (PBx) have gained popularity and are recommended by guidelines^1–4^.

A recent systematic review evaluated comparative data between TR and TP targeted PBx while critically assessing the quality of published evidence comparing the two approaches^5^. Of the 3608 references identified, only 6 studies were included in their review. The authors concluded that good-quality evidence comparing MRI/TRUS fusion guided TP and TR is lacking. They also questioned whether future prospective randomized studies should be performed given the concern of increased infection, which is believed to be associated with the transrectal approach. They favored the use of prospective databases and comparison with historical TR biopsy cohorts^5^.

Following this pragmatic approach, we compared demographics, imaging features, periprocedural complications and histologic outcomes of a cohort of men who underwent TP MRI/TRUS fusion PBx with a pair-matched synchronous cohort undergoing TR MRI/TRUS fusion PBx.

## Materials and methods

### Ethical approval

This study was approved by the Institutional Review Board and Ethical Committee of the University of Southern California (IRB No. HS-13-00663). All procedures performed were in accordance with the ethical standards of the institutional and/or national research committee, the 1964 Helsinki Declaration and its later amendments, or comparable ethical standards. Informed consent was obtained from all subjects and/or their legal guardian(s).

### Study population

Consecutive men who underwent prostate MRI followed by PBx at the University of Southern California between January 2017 and July 2021 were identified from a prospectively maintained Institutional Review Board approved PBx database. The inclusion criteria for this study were: (I) men with 3 T multiparametric (mp) MRI (T2-weighted [T2W], diffusion-weighted imaging [DWI], apparent diffusion coefficient [ADC], and dynamic contrast-enhanced [DCE])^6^ within 6 months prior to PBx; (II) PCa treatment naïve. Exclusion criteria were: (I) men who underwent mpMRI longer than 6 months prior to biopsy; (II) prior treatment for PCa; (III) prior surgery for benign prostatic hyperplasia (IV) prior saturation PBx. (V) mpMRI that did not meet Prostate Imaging-Reporting and Data System (PIRADS) v.2.0^7^ or v.2.1^8^ standards, including artifacts or poor imaging quality. The inclusion and exclusion criteria were same for applied to TP and TR cohorts.

### MRI acquisition and imaging interpretation

Glucagon 1 mg IM was administered prior to mpMRI of the prostate. mpMRI was performed on a 3 T MRI scanner (MR-750, General Electric, USA) with a 16-channel phased-array surface coil. Sequences included (but were not limited to) small field of view axial T2W, DWI using b100, b800 and b1400, ADC map generated from b800, and DCE during the intravenous injection of 0.2 ml/kg gadobenate dimeglubine (MultiHance, Bracco Diagnostics, Germany) at 3 ml/s^6^. mpMRI was acquired and interpreted based on PIRADS version 2.0^7^ or 2.1^8^ according to the current version at the time of biopsy. MRIs acquired at outside institutions were accepted if they met PIRADS requirements and inclusion/exclusion criteria for the current study. Images were interpreted by experienced radiologists with more than 5 years of experience in reading prostate mpMRI. The lesion with the highest PIRADS score was defined as the index lesion. MRIs were reviewed by an experienced urologist (ALA) with more than 2000 MRI/PBx each. Any discrepancy in imaging and reports was further reviewed by an experienced radiologist (SP) with more than 15 years of experience reading mpMRI prostate^6,9^.

### Prostate biopsy protocol

**Figure 1:**
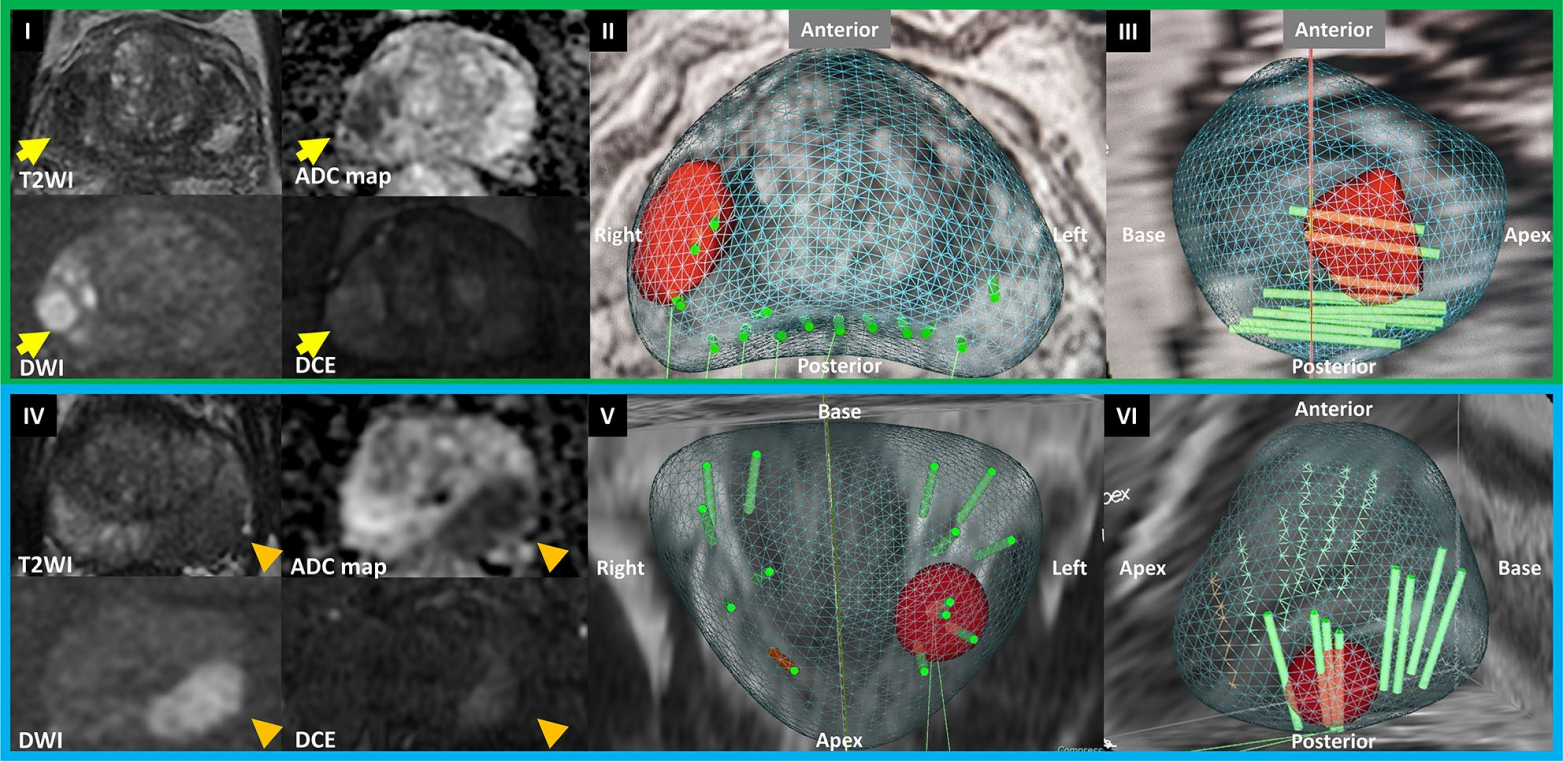
Representative images of transperineal and transrectal MRI/TRUS fusion prostate biopsy. (**I**–**III**) Transperineal MRI/TRUS fusion prostate biopsy (**I**) Pre biopsy mpMRI showing PIRADS score 5 lesion (yellow arrow) in the right mid peripheral zone. (**II**) Axial and (**III**) right sagittal view of Transperineal MRI/TRUS fusion prostate biopsy. (**IV**-**VI**) Transrectal MRI/TRUS fusion prostate biopsy. (**IV**) Pre biopsy mpMRI showing PIRADS score 5 lesion (orange arrowhead) in the left mid peripheral zone; (**V**) coronal view and (**VI**) left sagittal view of Transrectal MRI/TRUS fusion prostate biopsy. *MRI* magnetic resonance imaging, *TRUS* transrectal ultrasound, *mp* multiparametric, *PIRADS* prostate imaging reporting & data system, *T2WI*, *T2* weighted image, *ADC* apparent diffusion coefficient, *DWI* diffusion-weighted imaging, *DCE* dynamic contrast-enhanced.

Prostate biopsies were performed transperineally or transrectally, using a three-dimensional organ-tracking elastic image fusion system (Trinity, Koelis®, Grenoble, France) and 18G needle-biopsy, under local anesthesia or sedation, by a single urologist (ALA), as previously described (Fig. 1)^6,10–14^. All men underwent MRI followed by 12–14 core systematic biopsy (SB). In patients with prostate volume (PV) exceeding 50 cc, 14 systematic cores were obtained, whereas for smaller prostates (≤ 50 cc), 12 systematic cores were taken. Men with PIRADS scores 3–5 underwent at least two additional target biopsy (TB) cores per suspicious lesion. The PBx specimens were individually labeled and submitted in separate containers for uropathologist evaluation according to the International Society of Urological Pathology (ISUP) guidelines^15^. Empiric antibiotic prophylaxis was prescribed according to American Urological Association recommendations^16^. Patients undergoing TR PBx received 3 days of Ciprofloxacin, Bactrim or Cefuroxime with augmentation of Gentamicin IM prior to biopsy. Patients undergoing TP biopsy received a single dose of Cefuroxime^1,16^. Those with cardiac valve disease or replacement received additional injectable Gentamicin or Ceftriaxone prior to biopsy^11,16,17^.

### Definitions and endpoint

Men undergoing TP PBx were matched 1:2 with a synchronous cohort who underwent TR PBx by the following parameters: prostate specific antigen (PSA), PV and PIRADS score. The endpoint of the current study was the detection of clinically significant prostate cancer (CSPCa) on SB, TB, and SB + TB according to TP vs TR PBx approaches. Additionally, CSPCa was also reported as per lesion fashion. Demographics, imaging characteristics, and detailed PBx histologic findings were also analyzed.

The PCa and CSPCa detection outcomes on TB were presented as per index lesion defined as the highest PIRADS score, followed by the largest lesion on MRI. On MRI, the index lesion location within the prostate was defined as follows: anterior from 9 to 3:00 position, posterior from 3 to 9:00 position. Cases with that large lesions encompassed anterior and posterior location were assigned to both areas. Therefore, the sum of anterior and posterior area percentages could be more than 100 percent. The same methodology was applied to the lesion location at the base/mid/apex of the prostate.

CSPCa was defined as ISUP grade group (GG) 2 or greater^7,10^. PV was measured on MRI using ellipsoid formula (PV = height x width x length × 0.52). Patient’s race and ethnicity were self-assessed according to National Institutes of Health guidelines^18^. Complications were recorded up to 30 days post biopsy, according to Clavien-Dindo (CD) classifications^19^. Operative time was recorded from the moment of TRUS probe insertion to removal from the rectum.

### Transperineal versus transrectal approach

TP or TR approach were offered according to the risks of complications (infection and bleeding). The type of approach was not defined according to imaging findings or other parientscharacteristics. Patients with a high risk for infection or bleeding were offered TP PBx^16^. Other patients without specific indications for TP biopsy were offered either approach based on their preference. The patients were pair-matched as described above.

### Statistical analysis

For subgroup analysis, the patients were divided into two cohorts according to PIRADS score 1–2 (“negative MRI”) and 3–5 groups. The Wilcoxon rank sum test was used for continuous variables and the Fisher exact test was used for categorical variables. Logistic regression analysis was performed to identify clinical parameters related to CSPCa detection. Statistical analyses were carried out using SAS version 9.4 (SAS Institute Inc., NC, USA). A two-sided p value < 0.05 was considered statistically significant.

## Results

Overall, 504 patients met the inclusion criteria. A total of 168 TP PBx (Supplementary Fig. 1) were pair-matched to 336 TR PBx patients. The median age was 67 vs 66yrs, p = 0.3; PSA 7.46 vs 7.19 ng/mL, p = 0.9; PV 56 vs 52 cc, p = 0.5; PIRADS 1–2 (29% vs 29%), PIRADS 3 (21% vs 21%), PIRADS 4 (28% vs 31%) and PIRADS 5 (23% vs 19%), p = 0.8; lesion size on MRI 13 vs 13 mm, p = 0.3; and number of MRI lesions (1 vs 1), p = 0.2; were similar between TP vs TR PBx at baseline (Table 1), respectively. The index lesion location (anterior or posterior; base, mid or base) within the prostate were similar for TP vs TR groups (Table 1).

**Table 1:** Demographics of transperineal vs transrectal MRI/TRUS fusion prostate biopsy.

	All patients			MRI					
				PIRADS 1–2			PIRADS 3–5		
	Perineal	Rectal	p	Perineal	Rectal	p	Perineal	Rectal	p
No. of patients, n (%)	168 (33)	336 (67)		48 (33)	96 (67)		120 (33)	240 (67)	
Age, year, median (IQR)	67 (61–72)	66 (61–71)	0.3	65 (59–70)	64 (59–68)	0.7	67 (63–72)	67 (62–73)	0.4
Carlson comorbidity index, median (IQR)	1 (0–2)	1 (0–2)	0.5	1 (0–2)	1 (0–2)	0.3	1 (0–2)	1 (0–2)	1.0
Family history PCa, n (%)	45 (29)	87 (28)	0.9	22 (48)	18 (20)	0.001	23 (21)	69 (31)	0.052
Race, n (%)			0.5			0.7			0.3
Caucasian	98 (58)	204 (61)		26 (54)	63 (66)		72 (60)	141 (59)	
Black	7 (4.2)	17 (5.1)		3 (6.3)	6 (6.3)		4 (3.3)	11 (4.6)	
Latino	14 (8.3)	38 (11)		7 (15)	9 (9.4)		7 (5.8)	29 (12)	
Asian	18 (11)	30 (8.9)		6 (13)	9 (9.4)		12 (10)	21 (8.8)	
Other or not reported	31 (18)	47 (14)		6 (13)	9 (9.3)		25 (21)	38 (16)	
Biopsy history, n (%)			0.5			0.4			0.5
Naïve	102 (61)	193 (58)		29 (60)	46 (48)		73 (61)	147 (62)	
Negative	35 (21)	85 (25)		12 (25)	31 (32)		23 (19)	54 (23)	
In active surveillance	31 (18)	57 (17)		7 (15)	19 (20)		24 (20)	38 (16)	
PSA, ng/ml, median (IQR)	7.46 (5.21–10.68)	7.19 (5.00–10.66)	0.9	7.70 (5.98–9.73)	6.44 (4.92–9.60)	0.3	7.29 (5.05–11.00)	7.63 (5.10–11.54)	0.7
PSA density, ng/ml^2^, median (IQR)	0.13 (0.09–0.22)	0.14 (0.09–0.23)	0.8	0.11 (0.07–0.15)	0.11 (0.08–0.15)	0.9	0.16 (0.09–0.23)	0.16 (0.09–0.25)	0.6
Suspicion for PCa on DRE, n (%)	37 (22)	108 (32)	0.021	5 (10)	16 (17)	0.5	32 (27)	92 (67)	0.034
Clinical T stage, n (%)*			0.009			0.4			0.018
T1	77 (75)	123 (56)		17 (94)	24 (83)		60 (71)	99 (52)	
T2a	14 (14)	40 (18)		0 (0)	3 (10)		14 (16)	37 (19)	
T2b-c	5 (4.9)	23 (10)		1 (5.6)	1 (3.5)		4 (4.7)	22 (12)	
T3/T4	7 (6.8)	34 (15)		0 (0)	1 (3.5)		7 (8.2)	33 (17)	
Prostate volume, cc, median (IQR)	56 (36–76)	52 (36–76)	0.5	68 (45–101)	63 (44–88)	0.6	53 (35–71)	49 (35–69)	0.7
No. MRI lesions, median (IQR)	1 (0–1)	1 (0–2)	0.2	–	–	–	1 (1–2)	1 (1–2)	0.035
MRI index lesion location^†^, n (%)									
Anterior	–	–	–	–	–	–	51 (43)	91 (38)	0.4
Posterior	–	–	–	–	–	–	100 (83)	195 (81)	0.7
Base	–	–	–	–	–	–	41 (34)	104 (43)	0.11
Mid	–	–	–	–	–	–	86 (72)	164 (68)	0.5
Apex	–	–	–	–	–	–	49 (41)	117 (49)	0.18
MRI index lesion size^†^, mm, median (IQR)	–	–	–	–	–	–	13 (9–17)	13 (9–19)	0.3
PIRADS score, n (%)									
PIRADS 1–2	48 (29)	96 (29)	0.8	48 (29)	96 (29)	–	–	–	
PIRADS 3–5^†^	120 (71)	240 (71)		–	–		120 (100)	240 (100)	
PIRADS 3	35 (21)	70 (21)		–	–		35 (29)	70 (29)	0.6
PIRADS 4	47 (28)	105 (31)		–	–		47 (39)	105 (44)	
PIRADS 5	38 (23)	65 (19)		–	–		38 (32)	65 (27)	

*PIRADS* prostate imaging reporting and data system, *MRI* magnetic resonance imaging, *No*. number, *IQR* interquartile range, *PCa*, prostate cancer, *CSPCa* clinically significant PCa (Grade Group > 1), *DRE* digital rectal examination.

*DRE findings of a possible clinical stage in case prostate biopsy confirms cancer.

^**†**^Index lesion (highest PIRADS, then the largest lesion).

Details of PBx histologic outcomes are reported in Table 2. For PIRADS 1–2, PCa (40% vs 27%; p = 0.13) and CSPCa (2.1% vs 6.3%; p = 0.4) detection were similar for TP and TR PBx, respectively (Table 2). For PIRADS 3–5 lesions, PCa (72% vs 78%; p = 0.19) and CSPCa (59% vs 60%; p = 0.9) detection were similar for TP vs TR PBx, respectively. On a per lesion-based analysis, the CSPCa detection for PIRADS 3 (21% vs 16%; p = 0.4), PIRADS 4 (51% vs 44%; 0.8) and PIRADS 5 (76% vs 84%; 0.3) was similar for TP vs TR PBx, respectively. The median maximum PCa TB core length (11 vs 9 mm; p = 0.022) and percent involvement by cancer (83% vs 65%; p < 0.001) were higher for TP vs TR PBx.

**Table 2:** Outcomes of transperineal vs transrectal MRI/TRUS fusion prostate biopsy.

	MRI					
	PIRADS 1–2			PIRADS 3–5		
	Perineal	Rectal	p	Perineal	Rectal	p
No. of patients, n (%)	48 (29)	96 (29)		120 (71)	240 (71)	
Grade group			0.2			0.6
Benign	29 (60)	70 (73)		34 (28)	53 (22)	
1	18 (38)	20 (21)		15 (13)	43 (18)	
2	1 (2.1)	4 (4.2)		30 (25)	60 (25)	
3	0 (0)	1 (1.0)		14 (12)	35 (15)	
4	0 (0)	0 (0)		16 (13)	28 (12)	
5	0 (0)	1 (1.0)		11 (9.2)	21 (8.8)	
No. of TB cores taken, median (IQR)	–	–	–	5 (4–6)	4 (2–5)	< 0.001
No. of positive TB cores, median (IQR)	–	–	–	4 (3–5)	2 (1–4)	< 0.001
PCa detection SB + TB, N (%)	–	–	–	86 (72)	187 (78)	0.19
PCa detection SB, N (%)	19 (40)	26 (27)	0.13	63 (53)	175 (73)	< 0.001
PCa detection TB, N (%)	–	–	–	78 (65)	148 (62)	0.6
CSPCa SB + TB, N (%)	–	–	–	71 (59)	144 (60)	0.9
CSPCa SB, N (%)	1 (2.1)	6 (6.3)	0.4	44 (37)	123 (51)	0.01
CSPCa TB, N (%)	–	–	–	67 (56)	117 (49)	0.2
CSPCa TB per lesion, N (%)						
PIRADS 3	–	–	–	14/67 (21)	27/167 (16)	0.4
PIRADS 4	–	–	–	32/63 (51)	59/133 (44)	0.8
PIRADS 5	–	–		31/41 (76)	62/74 (84)	0.3
Maximum cancer core length SB + TB (mm), median (IQR)	–	–	–	11 (7–13)	9 (5–12)	0.025
Maximum cancer core length SB (mm), median (IQR)	2 (1–3)	4 (1–10)	0.069	8 (4–10)	6.5 (3–10)	0.4
Maximum cancer core length TB (mm), median (IQR)	–	–	–	11 (8–13)	9 (6–12)	0.022
Maximum cancer core percent SB + TB (%), median (IQR)	–	–	–	85 (60–95)	65 (30–90)	< 0.001
Maximum cancer core percent SB (%), median (IQR)	10 (10–20)	10 (5–50)	1.0	60 (30–80)	50 (20–75)	0.040
Maximum cancer core percent TB (%), median (IQR)	–	–	–	83 (60–95)	65 (40–90)	< 0.001

*PIRADS* prostate imaging reporting and data system, *MRI* magnetic resonance imaging, *No*. number, *IQR* interquartile range, *PCa* prostate cancer, *CSPCa* clinically significant PCa (grade group > 1), *SB* systematic biopsy, *TB* target biopsy.

Univariate logistic regression analysis showed that age, previous negative biopsy status, PSA, abnormal digital rectal examination (DRE) findings, PV, the number of MRI lesions, PIRADS 3–5, and the number of TB cores were significant predictors for CSPCa on PBx (Table 3). On multivariable logistic regression analysis, independent predictors for CSPCa detection were age, PSA, PV, abnormal DRE findings, and PIRADS 3–5. TP vs TR approaches were not predictors for CSPCa detection.

**Table 3:** Univariable and multivariable analyses for clinically significant prostate cancer detection on transperineal vs transrectal MRI/TRUS fusion prostate biopsy.

Variables	Univariate			Multivariate		
	OR	CI (95%)	p	OR	CI (95%)	p
Age, year	1.08	1.05–1.11	< 0.001	1.09	1.05–1.13	< 0.001
Family history PCa	0.82	0.54–1.23	0.3			
Biopsy history						
Previous negative biopsy vs naïve	0.37	0.23–0.59	0.003	0.61	0.33–1.11	0.056
Previous positive biopsy vs naïve	0.78	0.48–1.26	0.3	1.20	0.63–2.31	0.19
PSA, ng/ml	1.04	1.02–1.06	0.002	1.06	1.03–1.10	< 0.001
PSA density*, ng/ml^2^	1.00	1.00–1.01	0.2			
Race						
Black vs non-black	1.08	0.47–2.46	0.9			
Asian vs NH-white	0.64	0.33–1.18	0.3			
Hispanic vs HN-white	0.67	0.36–1.21	0.4			
Black vs NH-white	0.98	0.42–2.26	0.6			
Others vs NH-white	0.94	0.57–1.55	0.6			
DRE, suspicious vs non-suspicious	18.9	10.7–35.8	< 0.001	4.72	2.69–8.54	< 0.001
Prostate volume, cc	0.98	0.97–0.98	< 0.001	0.97	0.97–0.98	< 0.001
No. MRI lesions	2.18	1.77–2.73	< 0.001	0.82	0.58–1.14	0.2
MRI lesion size, mm	1.06	1.03–1.10	< 0.001			
PIRADS 3–5 vs PIRADS 1–2	29.0	14.2–70.1	< 0.001	37.9	13.8–121.1	< 0.001
No. TB cores taken	1.48	1.36–1.62	< 0.001			
Prostate biopsy approach TP vs TR	0.93	0.64–1.35	0.7			

*PIRADS* prostate imaging reporting and data system, *MRI* magnetic resonance imaging, *OR* odds ratio, *CI* confidence interval, *PCa* prostate cancer, *CSPCa* clinically significant PCa (Grade Group > 1), *DRE* digital rectal examination, *DRE* digital rectal examination, *NH* non-hispanic.

*PSA density was calculated per 0.01 unit.

Operative time was longer for the TP approach (22.5 vs 20 min; p < 0.001) (Table 4). The 30-day complications were similar between the groups (3.0% for TP group vs 1.2% for TR group; p = 0.17). While one patient in the TR PBx group experienced CD grade 4 urinary tract infection (UTI)/sepsis, the TP PBx patients did not experience any CD grade 3 or higher complications. Four patients in the TR PBx group were hospitalized to treat UTI. Only one patient in the TP PBx group needed 1 day of hospitalization for observation after PBx for a suspicious transient ischemic attack. Although 28% and 13% of patients in the TP group had a previous history of UTI and anticoagulation usage, respectively, neither UTI nor rectal bleeding was observed in this group.

**Table 4:** Perioperative outcomes and complications after transperineal and transrectal MRI/TRUS fusion prostate biopsy.

	Perineal	Rectal	p
Number of patients	168	336	**–**
Procedure time for TP vs TR PBx, minutes	22.5 (19–30)	20 (16.9–24)	< 0.001
Complications, n (%)			
Any	5 (3.0)	4* (1.2)	0.17
Prolonged rectal bleeding	0 (0)	0 (0)	–
Urinary tract infection	0 (0)	4 (1.2)	0.3
Urinary retention	3 (1.8)	1 (0.30)	0.11
Transient ischemic attack	1 (0.60)	0 (0)	0.3
Vasovagal reflex	1 (0.60)	0 (0)	0.3
Clavien grade, n (%)			
I	4 (2.4)	1 (0.30)	0.04
II	1 (0.60)	3 (0.89)	1.0
III	0 (0)	0 (0)	–
IV	0 (0)	1 (0.30)	1.0

*MRI* magnetic resonance imaging, *TRUS* transrectal ultrasound, *TP* transperineal, *TR* transrectal, *IQR* interquartile range.

*One transrectal biopsy case experienced urinary tract infection and retention.

## Discussion

There is a paucity of studies comparing TP vs TR MRI/TRUS PBx in the MRI era. Studies comparing TP vs TR systematic PBx without the use of prostate MRI, have shown that TP and TR approaches have similar diagnostic accuracy for PCa detection; however, the TP approach is associated with a lower risk of fever and rectal bleeding^20^. The main impact of MRI-directed targeted PBx is on improving CSPCa diagnosis over non MRI directed PBx; however, good quality studies comparing TP vs TR PBx are needed. The aim of this study is to report the outcomes of a pair-matched TP vs TR MRI/TRUS fusion PBx.

A recent meta-analysis evaluated the diagnostic accuracy of TP vs TR MRI/TRUS fusion PBx and showed similar sensitivity and specificity for detecting CSPCa via both approaches. However, there was substantial heterogeneity across the studies^21^. Rai et al. conducted a similar meta-analysis showing that the CSPCa detection using the transperineal approach was significantly higher than the transrectal approach. However, the authors quoted the outcomes as “very low” certainty of the evidence thus reinforcing the sparsity of data and the necessity for additional studies^5^.

Recently, Zattoni et al. conducted a study comparing CSPCa detection in patients with PIRADS score 3–5, who underwent TP (N = 3307) vs TR (N = 1936) MRI/TRUS fusion TB alone^22^. The study showed that CSPCa detection was higher for TP TB, and this was an independent predictor of CSPCa. Although this is a multicenter (Europe, China and Australia) study with a large cohort, and therefore the results could be generalizable, this should be interpreted with caution due to several limitations and excessive heterogeneity. In fact, the study period encompassed different PIRADS versions, and the cohorts weren’t pair-matched. Most important, the baseline characteristics were different between the TP vs TR groups. Furthermore, the PIRADS distribution was different between the groups, and the results weren’t reported as per lesion fashion^22^. They reported CSPCa detection of 49.1% vs 35.2% (p < 0.01) on TP TB vs TR TB, respectively. Herein, we report CSPCa detection on TP TB vs TR TB, alone, of 56% vs 49% (p = 0.2), respectively. When combined (SB + TB), we report a CSPCa detection of 59% for TP vs 60% for TR (p = 0.9). Therefore, the CSPCa detection on TR TB in this multicenter study might be lower rather than the detection rate being higher in the TP TB. Additionally, a significant amount of CSPCa might have been missed by omitting SB on these multicenter cohorts.

Ber et al. conducted a within-person noninferiority trial in men undergoing TP vs TR MRI/TRUS fusion PBx^23^. In 77 participants, GG ≥ 2 PCa detection was similar for TP TB vs TR TB (22/24 vs 16/24, p = 0.07), respectively. Similar to our results, they found that cancer core length and involvement were significantly higher in the TP TB. Koparal et al. conducted a multicenter study comparing CSPCa detection of TP and TR TB^24^. A total of 276 TP patients were matched to 508 TR patients by age, DRE, PSA density and PIRADS score. They found that both TP TB and TP 12-core SB were superior to TR TB and TR SB (27.5% vs 19.5%, p = 0.012 and 24.6% vs 16.3%, p = 0.006, respectively). Although CSPCa detection on TP was higher than TR PBx, it was lower than our results and those previously reported^23,24^. This study was limited due to the absence of baseline differences between the groups other than covariates used for propensity score matching^24^.

One of the concerns when comparing TP vs TR MRI/TRUS fusion PBx is that the location of the suspicious lesions may affect the histologic outcomes. In fact, apical or anterior lesions might be more suitable for the TP approach providing better sampling with greater detection of CSPCa than the TR PBx. Conversely, posterior lesions at the base might be better sampled by the TR approach due to the proximity of the probe and the needle to the lesions. In our study, the distribution of PIRADS scores, the number and size of the MRI lesions, and the distribution of the lesion location (anterior or posterior; apex, mid or base) were similar between TP and TR groups. PCa and CSPCa detection were similar between the groups; however, the PCa core length and percent were greater in the TP PBx group when compared to TR PBx. This could be attributed to the PCa distribution and the angle of the needle trajectory sampling. TP PBx has the capability to sample in parallel to the craniocaudal axis, and therefore obtain more homogeneous tissue from the peripheral zone where approximately 80% of Pca arise from^23,25,26^. In contrast, TR PBx sampling has a trajectory perpendicular to the craniocaudal axis, therefore collecting a mixture of tissue from the peripheral and transition zones. This discrepancy in sampling angles may have an impact on the superior sampling ability of TP PBx. Inadequate PCa tissue samplingcan result inoverdiagnosis and underdiagnosis, leading to inappropriate decision making. Consequently, the improved sampling characteristics of TP PBx may enhance patients’ diagnosis, selection for appropriate staging and treatment, ultimately impacting their prognosis.

It is debatable if patients with negative MRI (PIRADS 1–2) should undergo PBx^1–4^. In fact, the PROMIS trial showed a negative predictive value of MRI for GG $\ge 2$ of 76%^27^. In the setting of negative MRI, we follow the guidelines and PBx is performed by shared decision-making with the patient^1–4^. Some patients were on active surveillance (15% for TP and 20% for TR) in whom prostate biopsy is recommended by surveillance protocol even if MRI is negative^1,28^.

Patients undergoing TP PBx are expected to experience lower post-PBx infection rates than TR PBx^20^. We reported that the difference of post biopsy UTI between the TP vs TR groups was not significant. However, patients with a high risk for infection underwent TP PBx. Due to this selection, patients undergoing TR PBx had a lower risk of infection. Additionally, TR PBx patients received antibiotic augmentation according to American Urological Association recommendations^16^. Nevertheless, 1.2% of the TR PBx patients experienced post procedural UTI including CD grade 4 urosepsis, thus reinforcing the inherently higher risk of infection with transrectal prostate biopsy despite using augmented or targeted prophylaxis^29^. Recent randomized controlled trials showed the safety of TP PBx without antibiotic prophylaxis^30,31^. However, patients with an increased risk of infection were not enrolled in this trial. In our study, patients undergoing TP PBx had a higher risk for infection, therefore antibiotic prophylaxis was performed according to guidelines and as reported elsewhere^1,17^. Urinary retention is a complication related to PBx. In fact, urinary retention was observed in 1.8% after TP PBx and in 0.3% after TR PBx (p = 0.11). A recent systematic review showed no significant difference in urinary retention between TP vs TR PBx^5^.

Our study represents one of the largest cohorts from the United States comparing TP vs TR MRI/TRUS PBx. Although this was not a randomized clinical trial, the patients were pair-matched using synchronous cohorts. Although a matched cohort design could potentially reduce statistical power, we still had a sample size of up to 504 cases. A matched cohort design has several advantages such as controlling confounders, minimizing baseline differences and selection bias, improving statistical efficiency, and increasing the precision, thereby achieving more direct comparisons between the groups. Moreover, employing matching as a preprocessing technique for a regression model has been demonstrated to mitigate model dependence, minimize the likelihood of bias, and reduce variance in estimating causal effects compared to employing regression analysis using unprocessed raw data^32^. In order to adjust the differences of the known confounders (PIRADS, PSA, and PV) as preprocessing, matching was conducted^33^. In fact, the baseline characteristics including the location of the MRI suspicious lesions were similar between the groups. It is worth emphasizing that patients undergoing TP PBx were selected based exclusively on the risk of infection or bleeding. Despite our efforts to address potential biases, we acknowledge the possibility of selection bias inherent to the retrospective study design. While known and measured baseline characteristics were adjusted, unmeasured variables and unconscious bias may have influenced the decision-making process regarding the approach of PBx. We reported the detection of PCa and CSPCa of TB alone, SB alone and combined (SB + TB) as per patient and per lesion fashion, allowing for full appreciation of the outcomes. The present study is not a multicenter study that would increase the generalizability of the results. Conversely, all biopsies were performed at a single center, therefore decreasing heterogeneity. While the results may not be extrapolated to non-tertiary centers, this study was conducted uniformly, reinforcing the reliability of the outcomes. Until robust evidence comparing TP vs TR MRI/TRUS fusion PBx is cumulated, it might be premature to sentence that the TP approach provides better CSPCa detection than the TR approach.

## Conclusions

Transperineal MRI/TRUS fusion biopsy can be accurately performed using the free-hands technique. It provides similar clinically significant PCa detection rates compared to transrectal biopsy; however, with larger prostate cancer core length and percent of core involvement.

### Supplementary Information

Supplementary Figure 1.

## Data Availability

The datasets analyzed during the current study are available from the corresponding author upon reasonable request.
