# Supplementary material for: Transperineal vs transrectal magnetic resonance and ultrasound image fusion prostate biopsy: a pair-matched comparison
Source: Sci Rep. 2023 Aug 18;13:13457. doi: 10.1038/s41598-023-40371-7 (PMC10439224; doi:10.1038/s41598-023-40371-7)

Transperineal vs transrectal magnetic resonance and ultrasound image fusion prostate biopsy: a pair-matched comparison.

Masatomo Kaneko[1,2], Luis G. Medina[1], Maria Sarah L. Lenon[1,3], Sij Hemal[1], Aref S. Sayegh[1], Donya S. Jadvar[4], Lorenzo Storino Ramacciotti[1], Divyangi Paralkar[1], Giovanni E. Cacciamani[1,5], Amir H. Lebastchi[1], Bodour Salhia[6], Manju Aron[1,3], Michelle Hopstone[5], Vinay Duddalwar[5], Suzanne L. Palmer[5], Inderbir S. Gill[1], Andre Luis Abreu*[1,5]

Authors' affiliations:

[1] USC Institute of Urology, Center for Image-Guided Surgery, Focal Therapy and Artificial Intelligence for Prostate Cancer, Los Angeles, California, USA

[2] Department of Urology, Graduate School of Medical Science, Kyoto Prefectural University of Medicine, Kyoto, Japan

[3] Department of Pathology, Keck School of Medicine, University of Southern California, Los Angeles, California, USA

[4] Dornsife School of Letters and Science, University of Southern California, Los Angeles, California, USA

[5] Department of Radiology, Keck School of Medicine, University of Southern California, Los Angeles, California, USA

[6] Department of Medicine and Translational Genomics, Keck School of Medicine, University of Southern California, Los Angeles, California, USA

***Corresponding author**:

Andre Luis Abreu, MD.

1441 Eastlake Ave, Suite 7416, Los Angeles, California 90089.

Phone: +1-323-865-3700, Fax: +1-323-865-0120,

E-mail: andre.abreu@med.usc.edu

Supplementary materials

Supplementary Fig. 1: Flowchart of Transperineal approach cohort selection

TP, Transperineal; PBx, prostate biopsy; BPH, benign prostatic hyperplasia; MRI, magnetic resonance imaging.

Supplementary Fig. 1

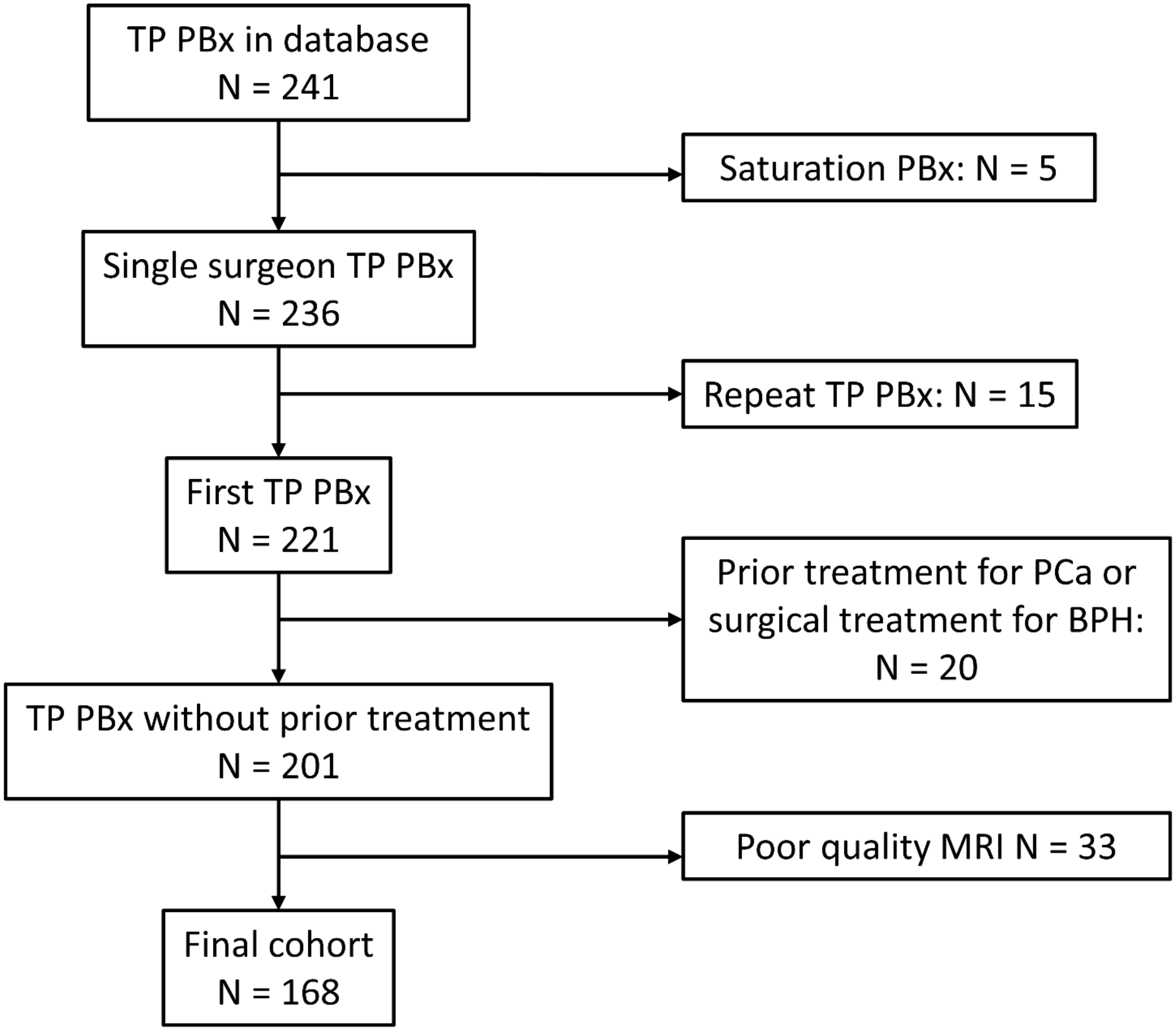

Supplement: Supplementary file 1 — Supplementary Figure 1. [file 41598_2023_40371_MOESM1_ESM.pdf]
